# Colorectal Cancer and Serum C-reactive Protein Levels: a Case-control Study Nested in the JACC Study

**DOI:** 10.2188/jea.15.S185

**Published:** 2005-08-18

**Authors:** Yoshinori Ito, Koji Suzuki, Koji Tamakoshi, Kenji Wakai, Masayo Kojima, Kotaro Ozasa, Yoshiyuki Watanabe, Miyuki Kawado, Shuji Hashimoto, Sadao Suzuki, Sinkan Tokudome, Hideaki Toyoshima, Norihiko Hayakawa, Kazuo Kato, Makoto Watanabe, Yoshiji Ohta, Morito Maruta, Akiko Tamakoshi

**Affiliations:** 1 Department of Public Health, Fujita Health University School of Health Sciences.; 2 Department of Public Health/Health Information Dynamics, Nagoya University Graduate School of Medicine.; 3 Division of Epidemiology and Prevention, Aichi Cancer Center Research Institute.; 4 Department of Health Promotion and Preventive Medicine, Nagoya City University Graduate School of Medical Sciences.; 5 Department of Epidemiology for Community Health and Medicine, Kyoto Prefectural University of Medicine Graduate School of Medical Science.; 6 Department of Hygiene, Fujita Health University School of Medicine.; 7 Department of Epidemiology, Research Institute for Radiation Biology and Medicine, Hiroshima University.; 8 Department of Pathology, Fujita Health University School of Health Sciences.; 9 Department of Internal Medicine, Fujita Health University School of Medicine.; 10 Department of Chemistry, Fujita Health University School of Medicine.; 11 Department of Surgery, Fujita Health University School of Medicine.; 12 Department of Preventive Medicine/Biostatistics and Medical Decision Making, Nagoya University Graduate School of Medicine.

**Keywords:** C-Reactive Protein, Colorectal Neoplasms, Odds Ratio, nested case-control studies

## Abstract

BACKGROUND: Recently, it has been hypothesized that inflammation increases the risk of colorectal cancer. We investigated whether serum levels of C-reactive protein (CRP), a biomarker of inflammation, are associated with colorectal cancer, using serum samples collected in the Japan Collaborative Cohort Study (JACC Study).

METHODS: We conducted a nested case-control study in the JACC Study, investigating the relationship between the risk for colorectal cancer and serum levels of CRP determined by a high-sensitivity CRP enzyme immunoassay. The subjects recruited were 141 patients with colorectal cancer (63 males and 78 females) and 327 controls with no history of cancer (148 males and 179 females). Each case of colorectal cancer was matched for sex, age and participating institution to 2 or 3 controls. We used t-test to analyze mean differences in CRP levels between colorectal cancer cases and controls. Odds ratios (ORs) and their 95% confidence intervals (CIs) were calculated using a conditional logistic regression model after adjusting for the potential confounding factors.

RESULTS: Serum CRP levels were not clearly associated with the risk of colorectal cancer. The OR of the highest serum CRP levels was 1.18 (95% CI: 0.68-2.06) for colorectal cancer and 1.42 (95% CI: 0.73-2.74) for colon cancer, compared to subjects with lowest serum levels. The OR for incidence of colorectal cancer showed a similar trend, but the difference was not significant. Thus, high serum CRP levels did not appear to increase the risk of colorectal cancer.

CONCLUSIONS: The present results suggest that high serum CRP levels are not associated with the risk of colorectal cancer in the JACC Study.

Recently, the incidence of colorectal cancer, which is closely related to lifestyle factors, such as diet, exercise, smoking, and alcohol consumption, has increased for both sexes.^1^^-^^3^ Consistent with the hypothesis that inflammation increases the risk of colon cancer,^4^^,^^5^ administration of aspirin or non-steroidal anti-inflammatory drugs, has been shown to decrease the risk of colorectal cancer.^6^^,^^7^ Also, it has been reported that serum levels of C-reactive protein (CRP), which is associated with inflammation and synthesized by hepatocytes during acute inflammation,^8^^,^^9^ are elevated in persons who subsequently develop colon cancer.^10^^,^^11^

There have been no previous reports of the relationship between high serum CRP levels and the risk for colorectal cancer in a population-based cohort study of Japanese. In the present study, we used sera from the Japan Collaborative Cohort (JACC) Study to investigate whether high serum CRP levels are associated with the risk of colorectal cancer in Japanese.

## METHODS

### Subjects

The subjects in the JACC Study were 110,792 residents of 45 districts of Japan, ranging in age from 40 to 79 years.^12^ The colorectal cancer cases were defined as incident or deceased (International Statistical Classification of Diseases and Related Health Problem 10th Revision: C18, C19, and C20). Incident cases were recruited from 24 participating institutions: in 21 participating institutions, cases were followed-up from baseline to the end of 1997; in the other 3 participating institutions, cases were followed-up from baseline to the end of 1994, 1995, and 1996, respectively. Dead subjects were enrolled from 45 participating institutions, and were followed-up from baseline to the end of 1999.

Peripheral blood samples were collected from 39,242 subjects (about 35% of respondents to the questionnaire survey) and stored in deep freezers at about -80°C for 13 to 15 years. During follow-up, 76 deaths from colorectal cancer (50 colon and 26 rectum) and 185 incident cases of colorectal cancer (123 colon and 62 rectum) were identified among subjects who had provided serum samples at baseline; 25 of those subjects (23 cases with history of any cancer and 2 cases with lacking a serum sample) were excluded from this study. For each case of colorectal cancer, 2 or 3 controls were matched for sex, age (as near as possible), and participating institution from among the surviving subjects without incident cancer or history of cancer. The study subjects were 141 colorectal patients (63 males and 78 females) and 327 controls (148 males and 179 females); i.e., out of an initial enrollment of 236 cancer cases and 661 controls, we excluded 95 cases and 218 controls who lacked sufficient serum volume for CRP determination, and we exclude 116 controls who were not suitably matched to patients for sex, age and participating institution. The cases and controls had similar sex distributions, and the age distribution was narrower for subjects aged from 40 to 49 years than for the other age group (Table 1). Colorectal cancer cases and controls also had similar distributions of smoking and alcohol consumption.

**Table 1.** Baseline chracteristics of cases of colorectal cancer and controls.

Item		Cases (%)		Controls (%)	
Number		141	(100)	327	(100)

Sex	Males	63	(44.7)	148	(44.3)
	Females	78	(55.4)	179	(54.7)

Age (year)	40-49	12	(8.5)	29	(8.9)
	50-59	44	(31.2)	117	(35.8)
	60-69	60	(42.6)	128	(39.1)
	70-79	25	(17.7)	53	(16.2)

Smoking habit	Current smoker	30	(21.3)	79	(24.2)
	Former smoker	21	(14.9)	43	(13.1)
	Never smoker	83	(58.9)	182	(55.7)
	Unknown	7	(5.0)	23	(7.0)

Alcohol consumption	Current drinker	67	(47.5)	144	(44.0)
	Former drinker	1	(0.7)	9	(2.8)
	Never drinker	68	(48.2)	191	(58.4)
	Unknown	5	(3.5)	13	(4.0)

Informed consent for participation was obtained individually from study subjects, with the exception of participating institutions with few participants, in which case informed consent was provided at the group level after the aim of the study and confidentiality of the data had been explained to community leaders. The Ethics Committees of Medical Care and Research of Fujita Health University and Nagoya University School of Medicine approved the protocol of this study.

### Methods

Serum CRP levels were determined by enzyme immunoassay, using a high-sensitivity C-reactive protein enzyme immunoassay test kit (Diagnostic Automation Inc., Calabasas, CA, USA).^13^ Body mass index (BMI) was calculated as body weight (kg) divided by height (m) squared. All statistical analyses were performed using the Statistical Analysis System^®^ package. Geometric mean differences in serum CRP levels between cases and controls were examined using *t*-tests. Odds ratios (ORs) and their 95% confidence intervals (CIs) for colorectal cancer were calculated using a conditional logistic regression model. The ORs were computed to categorize cases according to the tertiles of serum CRP levels in the controls. Previous findings suggest that serum CRP levels are affected by daily smoking, daily alcohol consumption, and BMI.^13^ In the present study, we considered the potential confounding effects of smoking (never, former, or current smoker or unknown), alcohol consumption (never, former, current drinker or unknown), and BMI (continuous variable). To test for linear trends in OR over tertiles, each tertile was coded as 0, 1, or 2 and was then incorporated into conditional logistic regression models as a single variable. Two-side probabilities less than 0.05 were considered to indicate statistical significance.

## RESULTS

Geometric mean values of serum CRP levels did not significantly differ between colorectal cancer cases (0.43 mg/L; ranges of 25% and 75%: 0.20-110mg/L, n = 141) and controls (0.45 mg/L; ranges: 0.20-1.03mg/L, n = 327), and also did not significantly differ between incident cases (0.37mg/L; ranges: 0.20-1.05mg/L, n = 104) and controls (0.45mg/L; ranges: 0.19-1.03 mg/L, n = 251). There was no clear association between serum CRP levels and the risk of colorectal cancer. In addition, geometric mean serum CRP levels of colon cancer cases (0.49mg/L, ranges: 0.20-1.05 mg/L, n = 101) were nearly equal to those for controls (0.49 mg/L, ranges: 0.23-1.00 mg/L, n = 237). The OR of the highest serum CRP levels for colorectal cancer was 1.07 (95% CI: 0.64-1.78), compared to the subjects with the lowest serum levels (Table 2). Also, the OR of the highest serum CRP levels was 1.18 (95% CI: 0.68-2.06) after adjusting for additional confounding factors, compared to the subjects with the lowest serum levels. The OR of the highest serum CRP levels for incident cases of colorectal cancer was 0.97 (95% CI: 0.51-1.83) after adjusting for the confounding factors, compared to the subjects with the lowest serum levels. In addition, the OR of the highest serum CRP levels was 1.42 (95% CI: 0.73-2.74) for colon cancer cases or 0.90 (95% CI: 0.30-2.30) for rectal cancer cases, compared to the lowest serum levels, but the difference was not significant. Higher serum CRP levels were not apparently associated with higher risk for colorectal cancer.

**Table 2.** Odds ratio (OR) and 95% confidence interval (CI) for colorectal cancer risk by serum C-reactive protein (CRP) level for the nested case-control study in the JACC Study.

Study cases	Ranks for serum CRP level	Ranges (mg/L)			No.		Crude			Adjusted*		
		
					Cases	Controls	OR	95% CI	p for trend	OR	95% CI	p for trend
Overall for colorectal cancer	T1 (lowest)		-	0.26	47	110	1.00	(reference)		1.00	(reference)	
	T2	0.27	-	0.80	46	107	1.00	0.60-1.66		1.13	0.66-1.94	
	T3 (highest)	0.81	-		48	110	1.07	0.64-1.78	0.80	1.18	0.68-2.06	0.57

Incidence for colorectal cancer	T1 (lowest)		-	0.26	37	84	1.00	(reference)		1.00	(reference)	
	T2	0.27	-	0.80	35	84	0.93	0.52-1.65		1.05	0.57-1.94	
	T3 (highest)	0.81	-		32	83	0.91	0.50-1.66	0.77	0.97	0.51-1.83	0.98

Overall for colon cancer	T1 (lowest)		-	0.26	34	79	1.00	(reference)		1.00	(reference)	
	T2	0.27	-	0.80	30	78	0.90	0.50-1.62		1.09	0.57-2.07	
	T3 (highest)	0.81	-		37	80	1.16	0.64-2.08	0.61	1.42	0.73-2.74	0.29

*: Adjusted for smoking and alcohol consumption, and body mass index.

## DISCUSSION

The JACC Study for Evaluation of Cancer Risk, sponsored by the Ministry of Education, Science, Sports, and Culture of Japan (Monbusho), was conducted to collect information from 110,792 subjects and sera from 39,242 subjects, aged from 40 to 79 years, in 45 districts of Japan from 1988 to 1990.^12^ In the present study, we investigated effects of serum CRP, which is a biomarker of inflammation and has recently been suggested to play a role in risk of colorectal cancer, using sera collected in the JACC Study. It has previously been shown that CRP is an acute phase protein that is produced as the result of activity of interleukin-6 (IL-6), IL-8, and tumour necrosis factors (TNF).^8^^,^^14^^,^^15^ Prostaglandins including prostaglandin E_2_ (PGE_2_) are synthesized in an arachidonic acid cascade initiated by cycloxygenase-2 (COX-2) enzyme, production of which is induced by inflammation.^15^^,^^16^ As levels of CRP have been shown to reliably predict cardiovascular events,^17^ and CRP and IL-6 have been shown to be associated with total and non-cardiovascular mortality,^18^^-^^20^ it has also been hypothesized in the review that inflammation can increase the risk of cancer.^21^ Recently, it has been reported that plasma CRP concentrations are elevated in subjects who subsequently developed and curative resection of colorectal cancer^5^^,^^10^^,^^11^^,^^22^ and that PGE_2_ levels appear to serve as a predictor of tumor recurrence inpatients with colorectal cancer.^23^ Moreover, evidence indicates that the selective effects of PGE_2_ consist of inhibiting secretion of IL-2, TNF-*β* and interferon gamma (IFN-*γ*) by helper T-1 (Th1) cells, and inhibiting secretion of IL-3 without affecting the secretion of IL-4 and IL-5 by helper T-2 (Th2) cells.^15^

It has been reported that the geometric mean values of plasma CRP concentrations were significantly higher for 172 colorectal incident cases (2.49mg/L) than for 342 controls (1.96mg/L; p = 0.01).^5^ Although the assay for serum CRP determination was different from that of the present study, serum CRP levels were not significantly higher for colorectal cancer cases than for controls. This is similar to the present findings for colon cancer cases, which the odds ratio of the highest serum CRP levels was 1.4 for colon cancer and 0.9 for rectal cancer, compared to the subjects with the lowest serum CRP levels. Higher serum CRP levels were not clearly associated with increased risk of colorectal or colon cancer.

As serum CRP levels have previously been shown to be associated with acute inflammation,^8^^,^^9^^,^^11^^,^^22^ the present lack of positive associations between higher serum CRP levels and increased risk of colorectal cancer may be due in part to the population-based method used in the present follow-up study. In addition, this lack of positive association may be due to sampling bias of serum CRP levels caused by the differences in serum treatment among the 45 study districts of the JACC Study. The present findings also differ from those of exactly matched case-control study, possibly due to the limited number of analytical cases at each participating institution, because of the high frequency of serum sample volumes that are inadequate for serum CRP measurement and the impossibility of creating matched case-control pairs at all participating institution. Also, the present data suggests that incidence cases of colorectal cancer have a slightly different trend in odds ratio from that of the overall colorectal cancer cases. Further studies, in which a distinction is made between incident cases of colorectal cancer and death from colorectal cancer, are needed to clarify the relationship between high serum CRP levels and the risk for colorectal cancer.

## MEMBER LIST OF THE JACC STUDY GROUP

The present investigators involved, with the co-authorship of this paper, in the JACC Study and their affiliations are as follows: Dr. Akiko Tamakoshi (present chairman of the study group), Nagoya University Graduate School of Medicine; Dr. Mitsuru Mori, Sapporo Medical University School of Medicine; Dr. Yutaka Motohashi, Akita University School of Medicine; Dr. Ichiro Tsuji, Tohoku University Graduate School of Medicine; Dr. Yosikazu Nakamura, Jichi Medical School; Dr. Hiroyasu Iso, Institute of Community Medicine, University of Tsukuba; Dr. Haruo Mikami, Chiba Cancer Center; Dr. Yutaka Inaba, Juntendo University School of Medicine; Dr. Yoshiharu Hoshiyama, Showa University School of Medicine; Dr. Hiroshi Suzuki, Niigata University School of Medicine; Dr. Hiroyuki Shimizu, Gifu University School of Medicine; Dr. Hideaki Toyoshima, Nagoya University Graduate School of Medicine; Dr. Shinkan Tokudome, Nagoya City University Graduate School of Medical Science; Dr. Yoshinori Ito, Fujita Health University School of Health Sciences; Dr. Shuji Hashimoto, Fujita Health University School of Medicine; Dr. Shogo Kikuchi, Aichi Medical University School of Medicine; Dr. Akio Koizumi, Graduate School of Medicine and Faculty of Medicine, Kyoto University; Dr. Takashi Kawamura, Kyoto University Center for Student Health; Dr. Yoshiyuki Watanabe, Kyoto Prefectural University of Medicine Graduate School of Medical Science; Dr. Tsuneharu Miki, Kyoto Prefectural University of Medicine Graduate School of Medical Science; Dr. Chigusa Date, Faculty of Human Environmental Sciences, Mukogawa Women’s University ; Dr. Kiyomi Sakata, Wakayama Medical University; Dr. Takayuki Nose, Tottori University Faculty of Medicine; Dr. Norihiko Hayakawa, Research Institute for Radiation Biology and Medicine, Hiroshima University; Dr. Takesumi Yoshimura, Institute of Industrial Ecological Sciences, University of Occupational and Environmental Health, Japan; Dr. Akira Shibata, Kurume University School of Medicine; Dr. Naoyuki Okamoto, Kanagawa Cancer Center; Dr. Hideo Shio, Moriyama Municipal Hospital; Dr. Yoshiyuki Ohno, Asahi Rosai Hospital; Dr. Tomoyuki Kitagawa, Cancer Institute of the Japanese Foundation for Cancer Research; Dr. Toshio Kuroki, Gifu University; and Dr. Kazuo Tajima, Aichi Cancer Center Research Institute.
